# Role of selected polymorphisms in determining muscle fiber composition in Japanese men and women

**DOI:** 10.1152/japplphysiol.00953.2017

**Published:** 2018-01-18

**Authors:** Hiroshi Kumagai, Takuro Tobina, Noriko Ichinoseki-Sekine, Ryo Kakigi, Takamasa Tsuzuki, Hirofumi Zempo, Keisuke Shiose, Eiichi Yoshimura, Hideaki Kumahara, Makoto Ayabe, Yasuki Higaki, Ryo Yamada, Hiroyuki Kobayashi, Akira Kiyonaga, Hisashi Naito, Hiroaki Tanaka, Noriyuki Fuku

**Affiliations:** ^1^Graduate School of Health and Sports Science, Juntendo University, Chiba, Japan; ^2^Japanese Society for the Promotion of Science, Tokyo, Japan; ^3^Faculty of Nursing and Nutrition, University of Nagasaki, Nagasaki, Japan; ^4^Faculty of Liberal Arts, The Open University of Japan, Chiba, Japan; ^5^Faculty of Medicine, Juntendo University, Tokyo, Japan; ^6^Faculty of Health and Nutrition, Tokyo Seiei College, Tokyo, Japan; ^7^Japan Institute of Sports Science, Tokyo, Japan; ^8^Department of Food and Health Sciences, Prefectural University of Kumamoto, Kumamoto, Japan; ^9^Faculty of Nutritional Sciences, Nakamura Gakuen University, Fukuoka, Japan; ^10^Faculty of Computer Science and Systems Engineering, Okayama Prefectural University, Okayama, Japan; ^11^Faculty of Sports and Health Science, Fukuoka University, Fukuoka, Japan; ^12^Center for Genomic Medicine, Kyoto University Graduate School of Medicine, Kyoto, Japan; ^13^Department of General Medicine, Mito Medical Center, Tsukuba University Hospital, Ibaraki, Japan

**Keywords:** ACE, ACTN3, myosin heavy chain isoform, polymorphism, sex difference

## Abstract

Genetic polymorphisms and sex differences are suggested to affect muscle fiber composition; however, no study has investigated the effects of genetic polymorphisms on muscle fiber composition with respect to sex differences. Therefore, the present study examined the effects of genetic polymorphisms on muscle fiber composition with respect to sex differences in the Japanese population. The present study included 211 healthy Japanese individuals (102 men and 109 women). Muscle biopsies were obtained from the vastus lateralis to determine the proportion of myosin heavy chain (MHC) isoforms (MHC-I, MHC-IIa, and MHC-IIx). Moreover, we analyzed polymorphisms in α-actinin-3 gene (*ACTN3*; rs1815739), angiotensin-converting enzyme gene (*ACE*; rs4341), hypoxia-inducible factor 1 α gene (rs11549465), vascular endothelial growth factor receptor 2 gene (rs1870377), and angiotensin II receptor, type 2 gene (rs11091046), by TaqMan single-nucleotide polymorphism genotyping assays. The proportion of MHC-I was 9.8% lower in men than in women, whereas the proportion of MHC-IIa and MHC-IIx was higher in men than in women (5.0 and 4.6%, respectively). Men with the *ACTN3* RR + RX genotype had a 4.8% higher proportion of MHC-IIx than those with the *ACTN3* XX genotype. Moreover, men with the *ACE* ID + DD genotype had a 4.7% higher proportion of MHC-I than those with the *ACE* II genotype. Furthermore, a combined genotype of *ACTN3* R577X and *ACE* insertion/deletion (I/D) was significantly correlated with the proportion of MHC-I (*r* = −0.23) and MHC-IIx (*r* = 0.27) in men. In contrast, no significant correlation was observed between the examined polymorphisms and muscle fiber composition in women. These results suggest that the *ACTN3* R577X and *ACE* I/D polymorphisms independently affect the proportion of human skeletal muscle fibers MHC-I and MHC-IIx in men but not in women.

**NEW & NOTEWORTHY** In men, the RR + RX genotype of the α-actinin-3 gene (*ACTN3*) R577X polymorphism was associated with a higher proportion of myosin heavy chain (MHC)-IIx. The ID + DD genotype of the angiotensin-converting enzyme gene (*ACE*) insertion/deletion (I/D) polymorphism, in contrast to a previous finding, was associated with a higher proportion of MHC-I in men. In addition, the combined genotype of these polymorphisms was correlated with the proportion of MHC-I and MHC-IIx in men. Thus *ACTN3* R577X and *ACE* I/D polymorphisms influence the muscle fiber composition in Japanese men.

## INTRODUCTION

Human skeletal muscles are composed of two main fiber types, namely, types I and II; type II muscle fibers are further divided into subgroups IIa and IIx (8). Type I fibers show high resistance to fatigue and are suitable for endurance performance, type IIa fibers are suitable for medium-term anaerobic exercise, and type IIx fibers are suitable for short bursts of strength and speed (5, 14). Type I fibers contain high levels of oxidative enzymes and low levels of glycolytic enzymes, whereas type IIx fibers contain high levels of glycolytic enzymes and low levels of oxidative enzymes; in contrast, the properties of type IIa fibers are intermediate to those of types I and IIx fibers (11). Simoneau and Bouchard (38) reported large, interindividual differences in the fiber-type composition of human skeletal muscle (i.e., 15–85% type I fibers, 5–77% type IIa, 0–44% type IIx) in healthy individuals. This variation in the composition of skeletal muscle fibers partly explains the marked difference in the physical performance of individuals, such as endurance running performance (32, 49), and occurrence of lifestyle-related diseases, such as obesity, type 2 diabetes mellitus, and hypertension (7, 15).

Genetic factors are suggested to play an important role in determining human skeletal muscle fiber composition. Komi et al. (18) reported for the first time that heritability estimates for muscle fiber composition were 99.5% in men and 92.8% in women. However, a sample size of that study was relatively small. Results of a previous study performed by Simoneau and Bouchard (37) showed that genetic factors (~45%) contributed more to the determination of the muscle fiber composition than environmental factors (~40%), with the remaining 15% because of muscle sampling and technical variance. These findings indicate that genetic factors exert a greater effect than environmental factors or that both of these factors exert comparable effects in determining muscle fiber composition.

Several studies have reported that some genetic polymorphisms, such as R577X (rs1815739) in the α-actinin-3 gene (*ACTN3*) (1, 47), insertion/deletion (I/D; rs4341) in the angiotensin-converting enzyme gene (*ACE*) (50), C/T polymorphism (rs11549465) in the hypoxia-inducible factor 1 α gene (*HIF1A*) (2), Q472H (rs1870377) in the vascular endothelial growth factor receptor 2 gene (*VEGFR2*) (3), and C/A polymorphism (rs11091046) in the angiotensin II receptor, type 2 gene (*AGTR2*) (25), are associated with muscle fiber composition. However, these findings are yet to be confirmed by other studies. Moreover, a sex-based difference has only been considered in one previous study (28).

Although many studies have shown sex-based differences in muscle fiber composition, conflicting reports are available on the proportion of fast and slow muscle fibers in men and women. Several studies have reported a higher proportion of type I fibers in women than in men (21, 38–40), whereas several studies have reported a higher proportion of type I fibers in men than in women (12, 17). Furthermore, several studies have reported no difference in the proportion of type I fibers between men and women (29, 34). These conflicting results may be associated with differences in sample size, age, ethnicity, and genetic background of study subjects included in these studies. Therefore, further studies are necessary to confirm the association between genetic polymorphisms and muscle fiber composition in men and women separately.

Therefore, the present study investigated the effects of five previously published genetic polymorphisms, namely, *ACTN3* R577X, *ACE* I/D, *HIF1A* C/T, *VEGFR2* Q472H, and *AGTR2* C/A, on muscle fiber composition with respect to sex-based differences in the general Japanese population.

## MATERIALS AND METHODS

### 

#### Subjects.

The present study included 211 Japanese subjects. The subjects were recruited from Juntendo University (30 men and 22 women; age 20−43 yr) and Fukuoka University (72 men and 87 women; age 21−79 yr). All of the subjects provided written informed consent before their inclusion in this study. The study protocols were approved by the Ethics Committees of Juntendo University and Fukuoka University.

#### Aerobic fitness.

Peak oxygen consumption (V̇o_2peak_) was measured using an incremental exercise test (15 W/min for men and 10 W/min for women) on a bicycle ergometer (Rehcor; Lode BV, Groningen, Netherlands). The test was continued until subjective exhaustion was achieved. We assumed that the participants had reached V̇o_2peak_ when at least two of the following criteria were met: *1*) a plateau in V̇o_2_ with an increase in the work load; *2*) blood lactate levels ≥8.0 mM; *3*) a respiratory exchange ratio ≥1.15; *4*) heart rate within 10 beats of the predicted maximum heart rate; and *5*) ratings of perceived exertion ≥19. Respiratory gas analysis was conducted using the mixing chamber method to evaluate the volume of expired air, and the O_2_ and CO_2_ fractions were analyzed by mass spectrometry (ARCO 1000 and 2000; Arco System, Chiba, Japan). Lactic acid was analyzed using the portable blood lactate analyzer (Lactate Pro; Arkray, Kyoto, Japan). To take into account individual differences in body weight, V̇o_2_ was expressed as kilograms of body weight.

#### Genotyping.

Total DNA was isolated from the venous blood of the study subjects by using the QIAamp DNA Blood Mini Kit (Qiagen, Hilden, Germany), according to the manufacturer’s instructions. The total DNA content was measured using a NanoDrop 8000 spectrophotometer (Thermo Fisher Scientific, Waltham, MA). Subsequently, DNA samples were adjusted to a concentration of 10 ng/μl with Tris-EDTA buffer and were stored at 4°C. *ACTN3* R577X (rs1815739), *ACE* C/G (I/D) (rs4341), *HIF1A* C/T (rs11549465), *VEGFR2* Q472H (rs1870377), and *AGTR2* C/A (rs11091046) polymorphisms were genotyped using a real-time thermocycler with an end-point analysis mode (LightCycler 480; Roche Applied Science, Mannheim, Germany) by using the TaqMan Single-Nucleotide Polymorphism (SNP) Genotyping Assay [assay identifications, *ACTN3* R577X: C_590093_1_, *ACE* C/G (I/D): C_29403047_10, *HIF1A* C/T: C_25473074_10, *VEGFR2* Q472H: C_11895315_20, *AGTR2* C/A: C_1841568_10]. A total of 5 μl of the genotyping mixture, containing 2.5 μl TaqMan GTXpress Master Mix (2×), 0.0625 μl TaqMan SNP Genotyping Assay (40×), and 1.4375 μl distilled water, was mixed with 1 μl genomic DNA (10 ng/μl) for each reaction. Four negative controls were included on each plate. Thermal cycling conditions included an initial denaturation at 95°C for 20 s, followed by 40 cycles of denaturation at 95°C for 3 s and annealing/extension at 60°C for 20 s. The *ACE* I/D genotype (rs4340) was determined using the *ACE* C/G genotype (rs4341), which is in perfect linkage disequilibrium with the I/D genotype as follows: C/C as II, C/G as ID, and G/G as DD (43). Allelic discrimination analysis was performed using LightCycler 480 software version 1.5.1.62 (Roche Applied Science). To confirm the accuracy of genotyping by the TaqMan SNP Genotyping Assay, we subjected at least 96 DNA samples, for which each genetic polymorphism sequence had been determined by direct sequencing to date. In each instance, the genotypes determined by the TaqMan SNP Genotyping Assay were identical to that determined by direct sequencing.

#### Muscle biopsy.

For this, 10–15 mg muscle samples were obtained from the belly of the vastus lateralis under sterile conditions and local anesthesia (1% lidocaine) by using a disposal needle-biopsy instrument (Max Core or Magnum; C.R. Bard, Covington, GA). The obtained muscle samples were frozen immediately in liquid nitrogen and were stored at −80°C until further analysis.

#### SDS-PAGE analysis of myosin heavy chain isoforms.

We assessed myosin heavy chain (MHC) isoforms as markers of the muscle fiber composition (35, 42, 44). The frozen muscle samples were homogenized in ice-cold lysis buffer [50 mM HEPES (pH 7.4), 10 mM EDTA, 4 mM EGTA, 50 mM β-glycerophosphate, 25 mM NaF, 5 mM Na_3_VO_4_, and 1% Triton X-100 or 10 mM HEPES, 70 mM sucrose, 220 mannitol, 100 mM KCl, 2 mM EDTA, 0.1% SDS, and 1% Nonidet P-40] containing a phosphatase inhibitor (PhosSTOP tablet; Roche Diagnostics, Indianapolis, IN) and a protease inhibitor (Complete tablet; Roche Diagnostics). The lysates obtained were centrifuged at 10,000 *g* for 10 min or 15,000 *g* for 60 min at 4°C. An insoluble pellet, obtained after homogenization, was suspended in a sufficient volume of SDS sample buffer [30% glycerol, 5% β-mercaptoethanol, 2.3% SDS, 0.05% bromophenol blue, and 62.5 mM Tris-HCl (pH 6.8)] and boiled at 95°C for 5 min. MHC composition was determined by performing glycerol SDS-PAGE, according to a method described previously (41), with some modifications. Briefly, protein samples were resolved by performing glycerol SDS-PAGE [stacking gel: 4% acrylamide, 34.7% glycerol, and 125 mM Tris-HCl (pH 6.8); separating gel: 8% acrylamide, 33.3% glycerol, and 375 mM Tris-HCl (pH 8.3)]. Electrophoresis was performed at 60 V and 8°C until the tracking dye exited the stacking gel and completely entered the separating gel. Voltage was set at 150 V, and electrophoresis was continued for 18 h at 8°C. Next, the gels were stained with Coomassie brilliant blue (Biosafe G250; Bio-Rad Laboratories, Hercules, CA) and were rinsed repeatedly with water. Each gel was scanned using a calibrated densitometer (ChemiDoc Touch Imaging System; Bio-Rad Laboratories). Relative concentrations of MHC-I, MHC-IIa, and MHC-IIx were determined using the calibrated densitometer (ChemiDoc Touch Imaging System) and analytical software (Image Laboratory software version 5.2.1; Bio-Rad Laboratories).

#### Statistical analysis.

Shapiro-Wilk test was used to assess the normality of all parameters. Data are expressed as means ± SD. Hardy-Weinberg equilibrium testing was performed for each SNP. Differences in phenotypes between men and women and among groups with different genotypes were analyzed using unpaired *t*-test and one-way ANOVA. Independent correlates of each muscle fiber composition were examined by performing multiple linear regression analysis. The combined effect of *ACTN3* R577X and *ACE* I/D polymorphisms was analyzed using Pearson’s correlation coefficient. Statistical significance was set at *P* < 0.05 for all comparisons. Statistical analyses were performed using JMP Pro version 12 (SAS Institute, Cary, NC).

## RESULTS

Men and women included in the present study did not show any significant difference with respect to age but showed significant differences in height body mass, body mass index (BMI), and V̇o_2peak_ (Table 1). The relative proportion of MHC-I was significantly lower in men than in women (40.5 ± 11.7 vs. 50.3 ± 11.1%, *P* < 0.001), whereas the relative proportion of MHC-IIa (35.8 ± 8.3 vs. 30.8 ± 8.2%, *P* < 0.001) and MHC-IIx (23.6 ± 9.2 vs. 19.0 ± 8.3%, *P* < 0.001) was significantly higher in men than in women. The correlations for each subject’s physical characteristics and muscle fiber composition in men and women are shown in Table 2. In men, age was significantly correlated with the proportion of MHC-I (*r* = 0.35, *P* < 0.001) and MHC-IIa (*r* = −0.36, *P* < 0.001). In women, age was significantly correlated with the proportion of MHC-I (*r* = 0.22, *P* = 0.023), height was significantly associated with the proportion of MHC-IIa (*r* = 0.26, *P* = 0.006), and body mass (*r* = 0.21, *P* = 0.032) and BMI (*r* = 0.24, *P* = 0.012) were significantly associated with the proportion of MHC-IIx.

**Table 1.** Characteristics of subjects

	Men (*n* = 102)	Women (*n* = 109)
Age, yr	46.7 ± 17.8	47.7 ± 16.5
Height, cm	169.7 ± 6.1	156.7 ± 5.9*
Body mass, kg	74.7 ± 11.5	63.9 ± 9.9*
BMI, kg/m^2^	25.9 ± 3.9	25.5 ± 4.3
V̇o_2peak_, ml⋅min^−1^⋅kg^−1^†	26.2 ± 6.1	23.3 ± 5.2‡
MHC-I, %	40.5 ± 11.7	50.3 ± 11.1*
MHC-IIa, %	35.8 ± 8.3	30.8 ± 8.2*
MHC-IIx, %	23.6 ± 9.2	19.0 ± 8.3*

BMI, body mass index; MHC, myosin heavy chain; V̇o_2peak_, peak oxygen consumption. Data are expressed as means ± SD.

* *P* < 0.001 vs. men.

† Data are available in 53 and 64 in men and women, respectively.

‡ *P* < 0.01 vs. men.

**Table 2.** Correlations (*r*) among each characteristic and muscle fiber composition in men and women

	MHC-I	MHC-IIa	MHC-IIx
Men			
Age, yr	0.35*	−0.36*	−0.13
Height, cm	−0.13	0.07	0.10
Body mass, kg	−0.08	−0.05	0.15
BMI, kg/m^2^	−0.03	−0.08	0.12
V̇o_2peak_, ml⋅min^−1^⋅kg^−1^	0.17	−0.04	−0.17
Women			
Age, yr	0.22†	−0.18	−0.12
Height, cm	−0.15	0.26*	−0.06
Body mass, kg	−0.12	−0.05	0.21†
BMI, kg/m^2^	−0.03	−0.16	0.24†
V̇o_2peak_, ml⋅min^−1^⋅kg^−1^	0.01	0.05	−0.06

BMI, body mass index; MHC, myosin heavy chain; V̇o_2peak_, peak oxygen consumption.

* *P* < 0.01.

† *P* < 0.05.

All of the polymorphisms followed the Hardy-Weinberg equilibrium. The rate of genotyping success was 211/211 (100%) for *ACTN3* R577X (rs1815739), 209/211 (99.1%) for *ACE* I/D (rs4340), 207/211 (98.1%) for *HIF1A* C/T (rs11549465), 208/211 (98.6%) for *VEGFR2* Q472H (rs1870377), and 209/211 (99.1%) for *AGTR2* C/A (rs11091046). Table 3 shows independent determinants of the composition of each muscle fiber in men and women. In men, the *ACE* I/D genotype was significantly associated with MHC-I, and the *ACTN3* R577X genotype was significantly associated with MHC-IIx. In contrast, no significant association was observed between muscle fiber composition and *HIF1A* C/T, *VEGFR2* Q472H, and *AGTR2* C/A polymorphisms. Moreover, no significant association was observed between muscle fiber composition and the analyzed genetic polymorphisms in women. Muscle fiber composition in each polymorphism, i.e., *ACTN3* R577X, *ACE* I/D, *HIF1A* C/T, *VEGFR2* Q472H, and *AGTR2* C/A, is shown in Table 4. Men with the *ACTN3* RR + RX genotype had a significantly higher proportion of MHC-IIx than men with the *ACTN3* XX genotype (24.8 ± 9.1 vs. 20.4 ± 8.6%, *P* = 0.031). Moreover, men with the *ACE* ID + DD genotype had a significantly higher proportion of MHC-I than men with the *ACE* II genotype (42.2 ± 10.8 vs. 37.6 ± 12.5%, *P* = 0.049).

**Table 3.** Independent determinants of muscle fiber composition

	MHC-I		MHC-IIa		MHC-IIx	
	β	*P*	β	*P*	β	*P*
Men						
Age, yr	**0.407**	**<0.001**	**–0.365**	**<0.001**	–0.191	0.074
BMI, kg/m^2^	–0.168	0.088	0.012	0.904	0.203	0.057
* ACTN3* genotype (RR = 0, RX = 1, XX = 2)	0.132	0.161	0.051	0.602	**–0.214**	**0.035**
* ACE* genotype (II = 0, ID = 1, DD = 2)	**0.221**	**0.021**	–0.123	0.209	–0.170	0.096
* HIF1A* genotype (CC = 0, CT = 1)	0.151	0.116	–0.153	0.124	–0.054	0.602
* VEGFR2* genotype (AA = 0, AT = 1, TT = 2)	–0.110	0.238	–0.026	0.791	–0.116	0.246
* AGTR2* genotype (CC = 0, CA = 1, AA = 2)	0.036	0.708	–0.057	0.561	0.007	0.949
Women						
Age, yr	**0.264**	**0.013**	–0.119	0.262	**–0.239**	**0.020**
BMI, kg/m^2^	–0.177	0.092	–0.113	0.287	**0.356**	**<0.001**
* ACTN3* genotype (RR = 0, RX = 1, XX = 2)	−0.076	0.442	0.084	0.410	0.019	0.848
* ACE* genotype (II = 0, ID = 1, DD = 2)	−0.064	0.508	0.042	0.669	0.045	0.634
* HIF1A* genotype (CC = 0, CT = 1)	0.093	0.344	0.052	0.599	–0.181	0.060
* VEGFR2* genotype (AA = 0, AT = 1, TT = 2)	0.159	0.110	–0.166	0.103	–0.047	0.623
* AGTR2* genotype (CC = 0, CA = 1, AA = 2)	–0.104	0.282	0.010	0.916	0.130	0.167

Covariates included in the multiple linear regression models were age, BMI, *ACTN3* R577X genotype (rs1815739), *ACE* I/D genotype (rs4340), *HIF1A* C/T genotype (rs11549465), *VEGFR2* Q472H genotype (rs1870377), and *AGTR2* C/A genotype (rs11091046). Values in bold denote *P* < 0.05.

**Table 4.** Muscle fiber composition by each genetic polymorphism in men and women

Gene name (rs number)	Genotype			*P*		
Men						
* ACTN3* (rs1815739)	RR (*n* = 24)	RX (*n* = 51)	XX (*n* = 27)	ANOVA	RR + RX vs. XX	RR vs. RX + XX
MHC-I, %	41.2 ± 12.0	39.3 ± 12.0	42.2 ± 10.9	0.547	0.382	0.736
MHC-IIa, %	35.2 ± 8.9	35.3 ± 8.1	37.4 ± 8.4	0.533	0.261	0.687
MHC-IIx, %	23.6 ± 6.7	25.4 ± 10.1	20.4 ± 8.6	0.069	**0.031**	0.952
* ACE* (rs4340)	II (*n* = 40)	ID (*n* = 48)	DD (*n* = 13)	ANOVA	II + ID vs. DD	II vs. ID + DD
MHC-I, %	37.6 ± 12.5	41.6 ± 9.8	44.6 ± 14.4	0.102	0.161	**0.049**
MHC-IIa, %	37.4 ± 8.9	35.1 ± 8.0	33.9 ± 8.1	0.309	0.374	0.143
MHC-IIx, %	25.0 ± 9.7	23.3 ± 8.1	21.4 ± 11.3	0.423	0.338	0.253
* HIF1A* (rs11549465)	CC (*n* = 90)	CT (*n* = 9)	TT (*n* = 0)	ANOVA	CC + CT vs. TT	CC vs. CT + TT
MHC-I, %	40.0 ± 12.0	46.2 ± 7.1				0.129
MHC-IIa, %	36.2 ± 8.4	31.8 ± 5.9				0.131
MHC-IIx, %	23.8 ± 9.5	22.0 ± 5.9				0.573
* VEGFR2* (rs1870377)	AA (*n* = 17)	AT (*n* = 42)	TT (*n* = 41)	ANOVA	AA + AT vs. TT	AA vs. AT + TT
MHC-I, %	36.8 ± 9.9	41.4 ± 12.3	40.9 ± 11.9	0.384	0.723	0.169
MHC-IIa, %	38.6 ± 8.0	34.4 ± 8.9	36.5 ± 7.8	0.188	0.573	0.163
MHC-IIx, %	24.6 ± 9.4	24.3 ± 8.6	22.6 ± 9.7	0.627	0.336	0.634
* AGTR2* (rs11091046)	CC (*n* = 66)	CA (*n* = 0)	AA (*n* = 35)	ANOVA	CC + CA vs. AA	CC vs. CA + AA
MHC-I, %	41.0 ± 11.4				39.3 ± 12.2	0.490
MHC-IIa, %	35.6 ± 8.2				36.5 ± 8.7	0.607
MHC-IIx, %	23.5 ± 9.2				24.3 ± 9.3	0.683
*Women*						
* ACTN3* (rs1815739)	RR (*n* = 27)	RX (*n* = 48)	XX (*n* = 34)	ANOVA	RR + RX vs. XX	RR vs. RX + XX
MHC-I, %	51.6 ± 13.1	49.3 ± 10.2	50.5 ± 11.0	0.696	0.889	0.471
MHC-IIa, %	30.6 ± 7.0	30.5 ± 7.2	31.3 ± 10.4	0.894	0.638	0.900
MHC-IIx, %	17.8 ± 9.4	20.2 ± 7.8	18.2 ± 8.0	0.392	0.508	0.396
* ACE* (rs4340)	II (*n* = 43)	ID (*n* = 48)	DD (*n* = 17)	ANOVA	II + ID vs. DD	II vs. ID + DD
MHC-I, %	50.9 ± 12.0	49.5 ± 10.3	49.6 ± 11.0	0.803	0.851	0.507
MHC-IIa, %	29.1 ± 7.8	32.9 ± 8.4	29.1 ± 8.0	0.060	0.369	0.084
MHC-IIx, %	20.0 ± 8.9	17.7 ± 7.7	21.3 ± 6.6	0.200	0.237	0.399
* HIF1A* (rs11549465)	CC (*n* = 95)	CT (*n* = 13)	TT (*n* = 0)	ANOVA	CC + CT vs. TT	CC vs. CT + TT
MHC-I, %	49.9 ± 11.2	51.1 ± 9.8				0.710
MHC-IIa, %	30.5 ± 8.4	32.6 ± 7.0				0.387
MHC-IIx, %	19.6 ± 8.0	16.2 ± 8.8				0.160
* VEGFR2* (rs1870377)	AA (*n* = 16)	AT (*n* = 53)	TT (*n* = 39)	ANOVA	AA + AT vs. TT	AA vs. AT + TT
MHC-I, %	48.0 ± 10.0	48.9 ± 10.5	52.6 ± 11.9	0.198	0.075	0.410
MHC-IIa, %	32.4 ± 7.2	31.4 ± 7.9	29.1 ± 9.0	0.289	0.126	0.380
MHC-IIx, %	19.6 ± 7.3	19.7 ± 8.6	18.3 ± 7.8	0.691	0.389	0.821
* AGTR2* (rs11091046)	CC (*n* = 39)	CA (*n* = 54)	AA (*n* = 15)	ANOVA	CC + CA vs. AA	CC vs. CA + AA
MHC-I, %	51.3 ± 12.4	50.3 ± 10.3	46.3 ± 9.4	0.325	0.151	0.398
MHC-IIa, %	30.6 ± 8.1	30.5 ± 8.5	31.9 ± 8.1	0.858	0.581	0.905
MHC-IIx, %	18.1 ± 9.3	19.2 ± 7.5	21.9 ± 6.3	0.311	0.163	0.308

Data are expressed as means ± SD. Data are available in 102 men in *ACTN3*, 101 men in *ACE*, 99 men in *HIF1A*, 100 men in *VEGFR2*, and 101 men in *AGTR2*. Data are available in 109 women in *ACTN3* and 108 women in *ACE*, *HIF1A*, *VEGFR2*, and *AGTR2*. Values in bold denote *P* < 0.05.

**Fig. 1.**
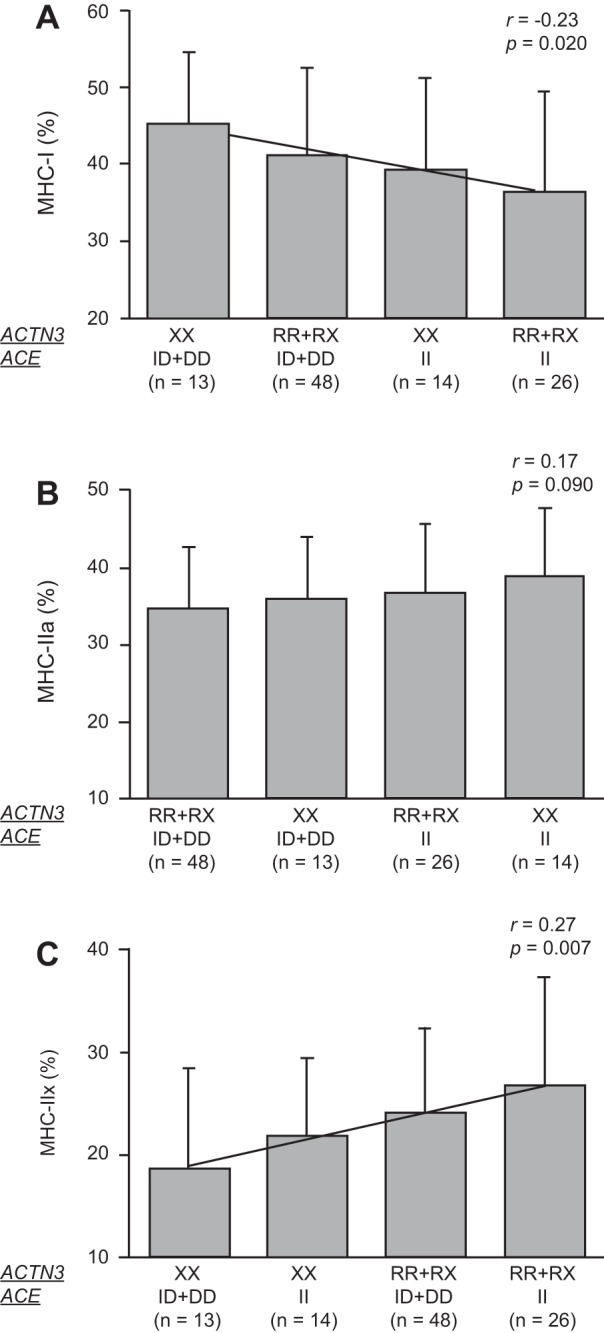
Most fitting models of combined effects of *ACTN3* R577X and *ACE* I/D on MHC-I (*A*), MHC-IIa (*B*), and MHC-IIx (*C*) in men. Data are expressed as means ± SD.

Figure 1 shows the most fitting models of combined effects of *ACTN3* R577X and *ACE* I/D polymorphisms on muscle fiber composition in men. In men, the *ACTN3* R577X and *ACE* I/D polymorphisms were significantly correlated with MHC-I (*r* = −0.23, *P* = 0.020; Fig. 1*A*) and MHC-IIx (*r* = 0.27, *P* = 0.007; Fig. 1*C*) but not MHC-IIa (*r* = 0.17, *P* = 0.090; Fig. 1*B*). Men with combined *ACTN3* XX and *ACE* ID + DD genotypes had the highest proportion of MHC-I and the lowest proportion of MHC-IIx, whereas men with combined genotypes of the *ACTN3* RR + RX and the *ACE* II had the lowest proportion of MHC-I and the highest proportion of MHC-IIx.

## DISCUSSION

In the present study, we investigated the effects of genetic polymorphisms in *ACTN3*, *ACE*, *HIF1A*, *VEGFR2*, and *AGTR2* on human skeletal muscle fiber composition with respect to sex-based differences in the general Japanese population. Our results showed that men with the *ACTN3* RR + RX genotype had a significantly higher proportion of MHC-IIx than those with the *ACTN3* XX genotype and that men with the *ACE* ID + DD genotype had a significantly higher proportion of MHC-I than those with the *ACE* II genotype. Results of multiple linear regression analyses showed that these effects remained significant after adjusting for living environmental factors, such as age and BMI. Furthermore, we observed the combined effects of both *ACTN3* R577X and *ACE* I/D polymorphisms on the proportion of MHC-I and MHC-IIx in men. However, no significant association was observed between muscle fiber composition and the examined genetic polymorphisms in women. Thus our results suggest that the *ACE* I/D and *ACTN3* R577X polymorphisms affect human skeletal muscle fibers MHC-I and MHC-IIx in men but not in women.

α-Actinins are important structural components of the Z-membrane, and expression of ACTN3 is limited to fast-twitch skeletal muscle fibers. A common nonsense polymorphism in *ACTN3* induces an amino acid substitution from arginine (R) to the stop codon (X) at position 577 (R577X), thus depleting the ACTN3 protein level in fast-twitch skeletal muscle fibers. Previous studies have shown that men with the *ACTN3* RR + RX genotype showed superior sprinting performance (20, 30). In the present study, we found that men with the *ACTN3* RR + RX genotype had a significantly higher proportion of MHC-IIx than men with the *ACTN3* XX genotype. This is consistent with the results of a study by Vincent et al. (47), which reported a higher proportion of MHC-IIx in the vastus lateralis of young, healthy men with the *ACTN3* RR genotype than those with the *ACTN3* XX genotype. However, this study did not assess the effect of the *ACTN3* RX genotype. In the present study, the proportion of MHC-IIx was higher in subjects with the *ACTN3* RX genotype than in those with the *ACTN3* XX genotype (*P* = 0.03; data not shown). Taken together, our results indicate that R-allele carriers (i.e., subjects with the RR and RX genotype) in the *ACTN3* R577X polymorphism had a higher proportion of MHC-IIx compared with subjects with the XX genotype in men but not in women.

The mechanism underlying the association between the *ACTN3* R577X polymorphism and muscle fiber composition may be associated with the signaling protein calcineurin. Calcineurin is a serine-threonine phosphatase activated by Ca^2+^-calmodulin, and calcineurin activation plays a key role in the determination and/or adaptation of slow-twitch muscle fibers (9, 10, 22, 23, 26). Previously, Seto et al. (36) have reported that calcineurin signaling was increased in ACTN3 knockout mice, and they also have demonstrated that human muscles of subjects with the XX genotype in the *ACTN3* R577X polymorphism showed significantly increased calcineurin signaling compared with subjects with the RR genotype. In the present study, we found that men with the *ACTN3* RR + RX genotype had a significantly higher proportion of MHC-IIx than those with the *ACTN3* XX genotype, which may be associated with changes in calcineurin signaling.

The *ACE* I/D polymorphism is one of the most common polymorphisms associated with physical performance. Several studies involving the European population have shown that the *ACE* I allele is associated with endurance performance, and the *ACE* D allele is associated with sprint/power performance (6, 13, 24, 27). In addition, a meta-analysis showed that the *ACE* I/I genotype is associated with endurance performance (19). However, almost all studies involving the Asian population have reported contrasting results (4, 16, 45, 48). We previously reported that the average running speed in a marathon was significantly higher in elite Japanese endurance runners with the *ACE* DD + ID genotype than those with the *ACE* II genotype (45). In addition, we reported that the *ACE* I allele was over-represented in elite short-distance Asian swimmers (48). These findings suggest that the *ACE* I/D polymorphism exerts different effects among different human ethnic groups. Interestingly, the present study showed that men with the *ACE* DD + ID genotype had a significantly higher proportion of MHC-I than those with the *ACE* II genotype, which is consistent with the results of previous studies involving the Asian population. However, Zhang et al. (50) reported conflicting results—that the *ACE* I allele was associated with a high proportion of MHC-I in 41 young Japanese subjects. Whereas the men to women ratio in the study by Zhang et al. (50) was different among the II, ID, and DD genotype groups, they did not consider the sex differences of the association between the *ACE* I/D polymorphism and muscle fiber composition. In the present study, we found that the proportion of MHC-I was higher in women than in men, suggesting that the conflicting results between the previous study by Zhang et al. (50) and the present study may be caused by sex differences in the muscle fiber composition. However, further studies are necessary to confirm our finding of the association between the *ACE* I/D polymorphism and muscle fiber composition in the Asian population.

Although it has been reported that the *HIF1A* rs11549465 (2), *VEGFR2* rs1870377 (3), and *AGTR2* rs11091046 (25) polymorphisms are associated with muscle fiber composition, this was not observed in the present study. Previous studies included young athletes and/or physically active, healthy men. However, the present study included subjects with a sedentary lifestyle who were comparatively older than those included in previous studies. This difference in study subjects may produce inconsistent results. Furthermore, we did not observe significant associations among the five genetic polymorphisms that we examined and muscle fiber composition in women. Previous studies have reported that estrogen, a female sex hormone, is associated with skeletal muscle growth, regeneration, and compositions (31, 33, 46). Thus estrogen may decrease the effects of the examined genetic polymorphisms on a proportion of MHC isoforms in women.

The present study has several limitations. The first limitation is the evaluation of muscle fiber composition. We only measured the proportion of MHC isoforms MHC-I, MHC-IIa, and MHC-IIx as an index of skeletal muscle fiber composition and did not measure the number and cross-sectional area of each fiber. The second limitation is the number of subjects. The present study included 211 subjects, which is much larger than the number of subjects included in similar, previous studies (1–3, 25, 47, 50). However, classification of the subjects, according to sex, relatively decreased the sample size. The third limitation is the problem of a multiple comparison when we analyze the associations between the selected genetic polymorphisms and MHC isoforms independently (Table 4). When we corrected the multiple comparisons in the present study, the statistical significances disappeared, likely because of the lack of statistical power of the present study. To avoid false negatives, we have shown statistical significance without adjustments for multiple comparisons. However, there is consequently an inflated possibility of false positives in the present study. Therefore, further and larger studies are needed to overcome the above limitations.

In the present study, we investigated the effects of genetic polymorphisms in *ACTN3*, *ACE*, *HIF1A*, *VEGFR2*, and *AGTR2* on the composition of human skeletal muscle fibers with respect to sex-based differences in the general Japanese population. Our results showed that men with the *ACTN3* RR + RX genotype had a significantly higher proportion of MHC-IIx than those with the *ACTN3* XX genotype and that men with the *ACE* ID + DD genotype had a significantly higher proportion of MHC-I than those with the *ACE* II genotype. Furthermore, results of multiple linear regression analysis showed that these effects remained significant even after an adjustment for living environmental factors, such as age and BMI. In contrast, no significant association was observed between muscle fiber composition and the examined genetic polymorphisms in women. Thus our results suggest that the *ACE* I/D and *ACTN3* R577X polymorphisms affect the proportion of human skeletal muscle fibers MHC-I and MHC-IIx in men but not in women.

## GRANTS

Support for this work was provided, in part, by grants from the Japan Society for the Promotion of Science (JSPS) Grants-in-Aid for Scientific Research B (KAKENHI; 15H03081 to N.F.) and the Ministry of Education, Culture, Sports, Science and Technology (MEXT)-Supported Program for the Strategic Research Foundation at Private Universities (to Juntendo University and Fukuoka University). H. Kumagai received a Grant-in-Aid for the JSPS Fellow from the JSPS.

## DISCLOSURES

No conflicts of interest, financial or otherwise, are declared by the authors.

## AUTHOR CONTRIBUTIONS

N.F. conceived and designed research; H. Kumagai, T. Tobina, N.I-S., R.K., T. Tsuzuki, K.S., E.Y., H. Kumahara, M.A., Y.H., H. Kobayashi, A.K., H.N., H.T., and N.F. performed experiments; H. Kumagai, H.Z., and R.Y. analyzed data; H. Kumagai and N.F. interpreted results of experiments; H. Kumagai prepared figures; H. Kumagai and N.F. drafted manuscript; H. Kumagai, T. Tobina, N.I-S., R.K., H.Z., R.Y., H.N., and N.F. edited and revised manuscript; N.F. approved final version of manuscript.
